# Cisto Hidático Cardíaco: Uma Causa Incomum de Bloqueio Atrioventricular Total

**DOI:** 10.36660/abc.20220597

**Published:** 2023-05-17

**Authors:** Renata Pibernat de Moraes, Mathias Silvestre de Brida, Rodrigo Moraes Reis, Raphael Santos Silva, Camila Bergonsi de Farias

**Affiliations:** 1 Instituto de Cardiologia Porto Alegre RS Brasil Instituto de Cardiologia, Porto Alegre, RS – Brasil

**Keywords:** Equinococose, Bloqueio Atrioventricular, Neoplasias Cardíacas

## Abstract

A hidatidose é uma zoonose causada pelo *Echinococcus granulosus*, levando à formação de cistos nos órgãos acometidos. O envolvimento cardíaco é raro e pode causar diversas complicações secundárias à ruptura, embolização ou compressão. Seu diagnóstico é desafiador, sendo confirmado por meio de dados relacionados a manifestações clínicas, exposição ambiental, exames laboratoriais e de imagem. A ressecção cirúrgica é necessária na maioria dos casos, sendo indicada a associação com terapia antiparasitária. No presente artigo, descreve-se um caso de cisto hidático cardíaco associado a bloqueio atrioventricular total em paciente jovem, com necessidade de implante de marcapasso, uma apresentação atípica e pouco relatada na literatura.

## Introdução

A hidatidose é uma doença parasitária endêmica causada pela forma larval do *Echinococcus granulosus*, infectando humanos acidentalmente por meio da ingestão de alimentos contaminados e resultando em formação de cistos. Os órgãos preferencialmente acometidos são fígado e pulmão, sendo o comprometimento cardíaco incomum (0,5 a 2%).^1^ Tendo em vista a maioria dos casos serem assintomáticos, o diagnóstico se torna desafiador.^2^ Descreve-se um caso de cisto cardíaco hidatiforme, de localização incomum, tendo como manifestação clínica o bloqueio atrioventricular (BAV) total em um paciente jovem, sendo, esta, uma associação de escassa descrição na literatura científica.

## Relato de Caso

Paciente de 38 anos, do sexo masculino, proveniente de região endêmica rural, previamente hígido e fisicamente ativo, procura atendimento por cansaço aos esforços há uma semana, com eventuais episódios de lipotimia. Negou uso de substâncias ilícitas, suplementos hormonais ou nutricionais proteicos, assim como tabagismo ou etilismo. Ao exame físico, apresentava-se em bom estado geral a despeito de bradicardia, com frequência cardíaca (FC) próxima de 35 bpm, sem sinais de instabilidade hemodinâmica ou alterações à ausculta cardiopulmonar. Não apresentava lesões dermatológicas ou outras anormalidades ao exame.

**Figura 1:**
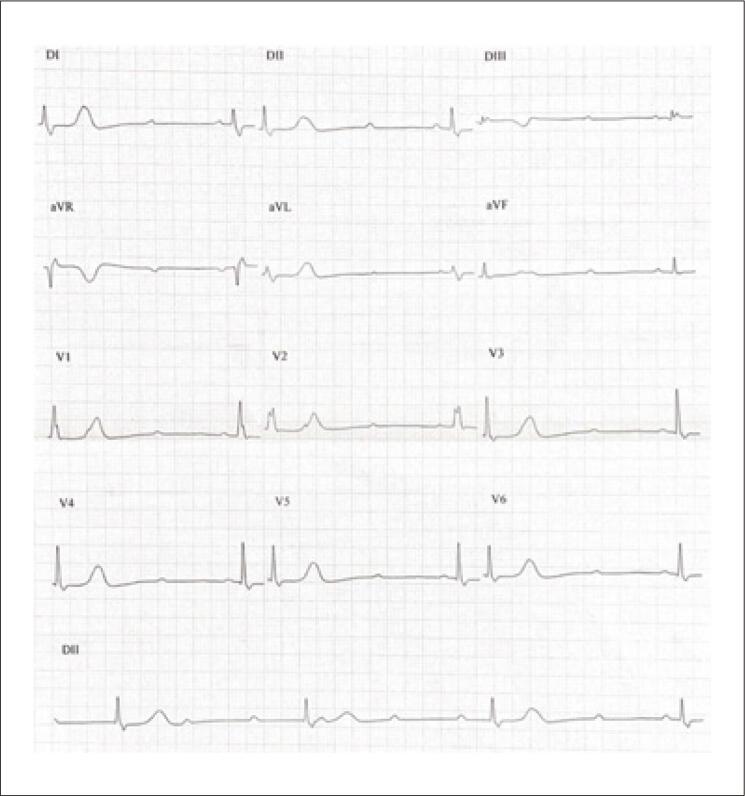
– Eletrocardiograma de 12 derivações demonstrando bloqueio atrioventricular total com ritmo de escape juncional e frequência cardíaca de 30 bpm.

Foi realizado eletrocardiograma (ECG), sendo identificado BAV total e ritmo de escape juncional, com FC de 30 bpm (Figura 1). Paciente permaneceu sob monitorização cardíaca contínua enquanto prosseguiu investigação diagnóstica. Exames laboratoriais demonstraram ausência de alterações metabólicas, endocrinológicas, inflamatórias ou de injúria miocárdica (Tabela 1). Foram realizadas sorologias para infecções crônicas, também não reagentes.

Fonte: o próprio autor.

**Tabela 1:** – Exames laboratoriais e valores de referência. Fonte: o próprio autor

Exames	Resultado	Valor de referência
Hemoglobina	13,0 g/dL	13,5-17,5 g/dL
Leucócito	10190 /mm^3^	3500-10500 /mm^3^
Eosinófilos	41 /mm^3^	50-500 /mm^3^
Plaquetas	269000 /mm^3^	140000-440000 /mm^3^
Magnésio	2,04 mg/dL	1,6-2,6 mg/dL
Potássio	4,4 mg/dL	3,4-5,4 mg/dL
Sódio	143 mg/dL	135-147 mg/dL
Cálcio iônico	4,67 mg/dL	4,48-5,2 mg/dL
TSH Ultrassensível	1,68 uUI/mL	0,27-4,20 uUI/mL
PCR Ultrassensível	0,51 mg/dL	<1,0 mg/dL
Glicose	102 mg/dL	70-100 mg/dL
pH arterial	7,44	7,35-7,45
HCO3 arterial	21 mmol/L	21-28 mmol/L
pCO2 arterial	32 mmHg	35-45 mmHg
Lactato	12,7 mg/dL	4,5-19,8 mg/dL
Creatinina	0,99 mg/dL	0,7-1,2 mg/dL
Ureia	39 mg/dL	16-50 mg/dL
Tropinina T Ultrassensível	9,77 pg/mL	< 14 pg/mL
Anti-HCV	NR*	NR*
Anti-HIV	NR*	NR*
HbsAg	NR*	NR*
Chagas	NR*	NR*
VDRL	NR*	NR*
FAN	NR*	NR*
Antígeno Sars-CoV-2	NR*	NR*
Anticorpo Hidatidose (IgM/IgG)	NR*	NR*

NR*: não reagente

**Figura 2:**
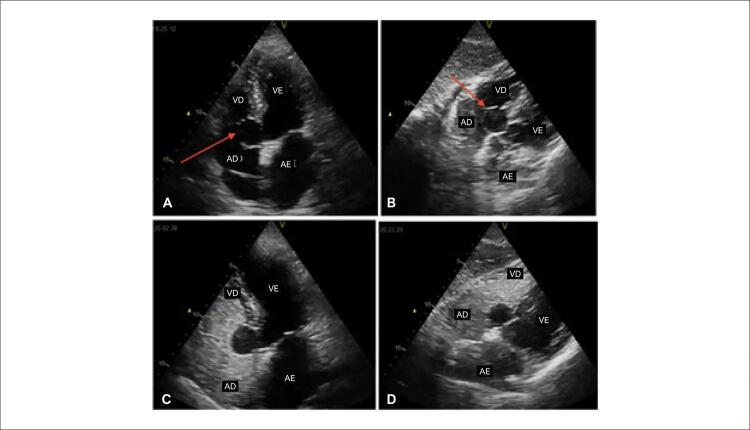
– Ecocardiograma demonstrando, em janela apical quatro câmaras (A) e subcostal (B), estrutura anecóica de aspecto cístico no interior do átrio direito, aderida ao septo interatrial e adjacente ao folheto septal tricúspide (setas vermelhas). Após a injeção periférica de solução salina agitada, observado um delineamento mais preciso da lesão, sem preenchimento desta pela solução (C e D). AE: átrio esquerdo; AD: átrio direito; VE: ventrículo esquerdo; VD: ventrículo direito.

Afastadas causas potencialmente reversíveis, optou-se por realizar ecocardiograma transtorácico (ETT), o qual demonstrou estrutura anecóica de aspecto cístico no interior do átrio direito, aderida ao septo interatrial e adjacente ao folheto septal tricúspide, sem causar eversão diastólica ou obstrução na via de entrada do ventrículo direito. Após a injeção periférica de solução salina agitada, observou-se delineamento mais preciso da estrutura, sem preenchimento desta ou passagem da solução agitada entre as câmaras (Figura 2).

Fonte: o próprio autor.

**Figura 3:**
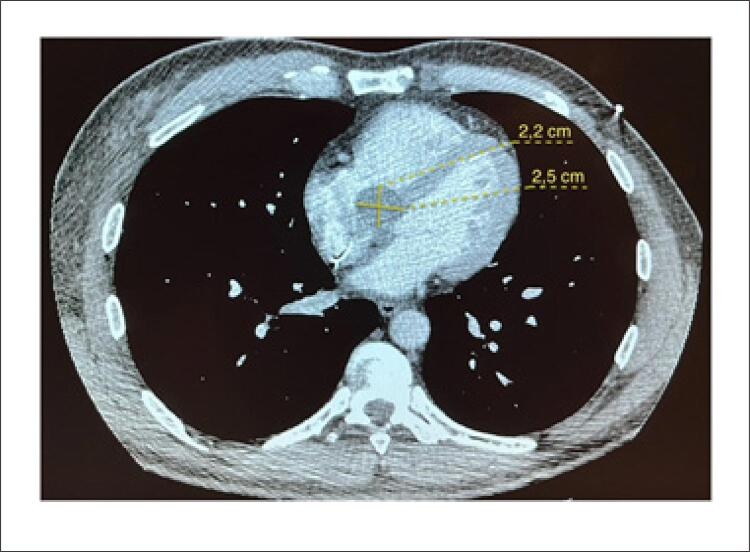
– Angiotomografia computadorizada de tórax em corte axial demonstrando imagem hipodensa de contornos regulares e lisos, medindo 2,5 x 2,2 cm, com conteúdo líquido, localizada próxima a região do septo interatrial e porção basal do septo interventricular.

Foram propostos cisto hidático ou cisto sanguíneo como hipóteses diagnósticas. De modo complementar, devido à indisponibilidade da ressonância magnética (RM), optou-se por realizar tomografia computadorizada (TC) de tórax e abdome para melhor caracterização da lesão e de seu conteúdo. Nesta, foi revelada imagem hipodensa de contornos regulares e lisos, medindo 2,5 x 2,2 cm, próxima à região basal do septo interventricular e septo interatrial, em cavidade atrial direita. A densidade era compatível com conteúdo líquido, sendo pouco provável ser sanguíneo mesmo após injeção de contraste. A TC de abdome não identificou cistos em outros órgãos (Figura 3).

Fonte: o próprio autor.

**Figura 4:**
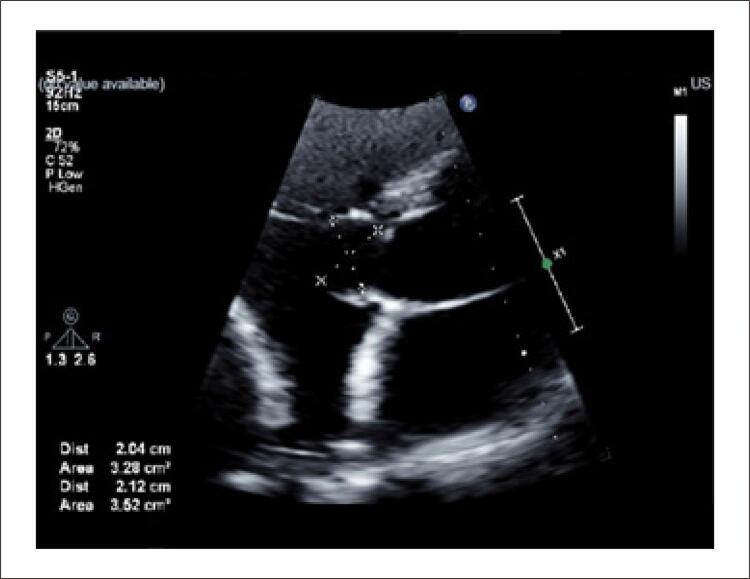
– Ecocardiograma transtorácico demonstrando imagem cística com discreta redução das dimensões em comparação com exame prévio, medindo aproximadamente 2,1 x 2,0 cm.

Apesar da sorologia negativa por imunofluorescência indireta, a exposição ambiental e os exames de imagens reforçaram a hipótese de cisto hidático e, de que este seja a causa do distúrbio de condução secundário à compressão extrínseca do nodo AV. Diante da recusa do paciente a se submeter ao procedimento cirúrgico nessa internação, mesmo ciente dos riscos, optou-se, em conjunto com familiares, por implante de marcapasso definitivo e posterior alta hospitalar com tratamento antiparasitário. Após três meses de seguimento ambulatorial, repetiu-se o ecocardiograma transtorácico, o qual demonstrou discreta redução das dimensões do cisto em comparação a imagem pré-tratamento medicamentoso (Figura 4) e sem sinais de complicações, permanecendo assintomático.

Fonte: o próprio autor.

## Discussão

A hidatidose, ou equinococose, é uma infecção parasitária geralmente causada pelo *Echinococcus granulosus*, ocorrendo de forma endêmica em algumas regiões do mundo. Quando em estágio larval, o parasita pode infectar humanos de forma acidental por meio da ingestão de alimentos contaminados, sendo os canídeos e outros carnívoros os hospedeiros definitivos e os onívoros e herbívoros os hospedeiros intermediários.^1^

A infecção comumente resulta em formação de cistos hepáticos e pulmonares, uma vez que a disseminação hematológica se dá através da absorção pelo trato gastrointestinal e consequente invasão da circulação portal e veia cava inferior.^3^ O comprometimento de outros órgãos é menos frequente, sendo o cardíaco extremamente incomum (0,5-2% dos casos). O acometimento ventricular esquerdo é o mais observado (60%), seguido pelo ventrículo direito (10%), pericárdio (7%), átrio esquerdo (6-8%) e septo interventricular (4%).^4^ O átrio direito, como no relato descrito, está envolvido apenas em 3-4% dos casos.^3^

As manifestações clínicas dependem da localização, crescimento e número de cistos. Podem se apresentar com sintomas inespecíficos (tosse, febre, perda de peso, fadiga, dor torácica) ou, na grande maioria, na forma assintomática.^5^ A hidatidose está associada a complicações como arritmias, eventos embólicos pulmonar ou sistêmica e choque anafilático secundário à ruptura.^3^ O crescimento do cisto pode causar complicações por compressão extrínseca como, por exemplo, isquemia miocárdica, distúrbios de condução, disfunção valvar ou obstrução de fluxos transvalvares.^1,6^

O diagnóstico é feito pela combinação de achados clínicos, testes sorológicos e exames de imagem.^3^ O teste de ELISA é um dos mais sensíveis para detectar anticorpos contra *Echinococcus granulosus*. No entanto, também podem-se usar testes de imunofluorescência indireta, fixação do complemento por imunoensaio enzimático (Weinberg) e teste de aglutinação em látex.^3,6^ Testes negativos não excluem o diagnóstico, pois a detecção seria maior em casos de disseminação hematogênica secundária a liberação do conteúdo do cisto. Com isso, métodos de imagem se tornam ferramentas indispensáveis para sua definição.^6^

O ETT é o método mais efetivo no diagnóstico devido ao fácil acesso e baixo custo. O cisto apresenta componente hipoecogênico e contorno regular e permite caracterizá-lo de forma detalhada quanto ao tamanho, quantidade, localização e identificação de complicações.^1^ A RM tem maior acurácia ao avaliar o conteúdo do cisto e sua relação com estruturas adjacentes.^3^ O aspecto é de lesão oval, hipointenso nas imagens ponderadas em T1 e hiperintenso em T2. Achado típico é um anel periférico hipointenso, representando pericisto (cápsula fibrosa densa do tecido hospedeiro reativo).^4^ Já na TC, observa-se estrutura hipodensa com acúmulo de contraste nas paredes.^6^ Um cisto hidático não complicado, como o caso relatado, mostra uma lesão homogênea bem definida, com baixa densidade e paredes lisas com espessura variável.^7^ Achados imagiológicos mais específicos incluem calcificação da parede do cisto, cistos filhos e descolamento de membrana.^3,4,7^

Quanto ao diagnóstico diferencial, pode-se considerar tumores cardíacos como mixomas, cistos sanguíneos e cisto pericárdico congênito.^4,7^ Neste caso relatado, a lesão cística foi identificada pelo ETT e sua análise complementada por TC, sendo hidatidose a principal hipótese.

A remoção cirúrgica é o tratamento de escolha mesmo em assintomáticos devido ao alto risco de ruptura, sendo necessário *bypass* cardiopulmonar para evitar disseminação.^6,8^ Após remoção, o tratamento de escolha é albendazol (10-15 mg/kg/dia) por 3 a 6 meses.^6,9^ Pacientes não candidatos ao tratamento invasivo devem usar o anti-helmíntico a fim de reduzir o crescimento do cisto.^6^ O tratamento pré-operatório tende a não ser indicado devido ao aumento da friabilidade da membrana, aumentando o risco de ruptura durante manipulação cirúrgica.^8^

O prognóstico pós-operatório é bom, com raras complicações maiores.^6,10^ Não há dados suficientes para os casos não submetidos ao procedimento cirúrgico. Apesar do procedimento ser considerado curativo na forma cardíaca isolada, possui risco de recorrência, necessitando acompanhamento a longo prazo.^6^

## Conclusão

A hidatidose é uma zoonose endêmica com raro acometimento cardíaco. Relatamos um caso de cisto hidático de localização incomum e apresentação clínica atípica, desencadeando bloqueio atrioventricular total em paciente jovem. O diagnóstico etiológico nesse cenário deve passar por ampla investigação, sendo os exames de imagem ferramenta essencial para o adequado manejo diagnóstico e definição terapêutica.
