# Supplementary material for: Cisto Hidático Cardíaco: Uma Causa Incomum de Bloqueio Atrioventricular Total
Source: Arq Bras Cardiol. 2023 May 17;120(5):e20220597. [Article in Portuguese] doi: 10.36660/abc.20220597 (PMC10348382; doi:10.36660/abc.20220597)
Supplement: Translation

# Translation: Cardiac Hydatid Cyst: An Uncommon Cause of Complete Atrioventricular Block

**Authors:** Renata Pibernat de Moraes, Mathias Silvestre de Brida, Rodrigo Moraes Reis, Raphael Santos Silva, Camila Bergonsi de Farias

**Affiliations:** 1 Brazil Instituto de Cardiologia, Porto Alegre, RS – Brazil

**Keywords:** Echinococcosis, Atrioventricular Block, Cardiac Tumors

## Abstract

Hydatidosis is a zoonosis caused by *Echinococcus granulosus*, leading to the formation of cysts on involved organs. Cardiac involvement is rare and can cause a wide range of complications secondary to rupture, embolization, or compression. Its diagnosis is challenging, and is generally confirmed through data related to clinical manifestations, environmental exposure, and laboratory and imaging exams. Surgical removal is necessary in most cases, in which an association with antiparasite therapy is recommended. The present article describes a case of a cardiac hydatid cyst associated with a complete atrioventricular block (AVB) in a young adult patient, with the need for a pacemaker implant, an atypical presentation, and scarce reports in the literature.

## Introduction

Hydatidosis is an endemic parasitic disease caused by the larva form of *Echinococcus granulosus*, infecting humans accidentally through the intake of contaminated food and resulting in the formation of cysts. The preferentially involved organs are the liver and the lungs, but cardiac involvement tends to be rare (0.5 to 2%).^1^ Given that most cases are asymptomatic, the diagnosis becomes rather challenging^2^ – describe a case of a hydatidform cardiac cyst, in an uncommon location, with a clinical manifestation of complete atrioventricular block (AVB) in a young adult patient, this being an association with scarce reports in the scientific literature.

## Case Report

A 38-year-old, male patient, from an endemic rural region, who was previously healthy and physically active, sought out medical care due to fatigue when exerting effort for a period of one week, with sporadic episodes of lipothymia. The patient denied any use of illicit substances, or hormonal or nutritional protein supplements; he was also a non-smoker and did not drink alcohol. Upon physical examination, he appeared to be in good health regarding bradycardia, with a heart rate (HR) near 35 bpm, with no signs of hemodynamic instability or alterations upon cardiopulmonary auscultation. His examination also presented no dermatological lesions nor other anomalies.

An electrocardiogram (ECG) was conducted, which identified a complete AVB and a junctional escape rhythm, with an HR of 30 bpm (Figure 1). The patient remained under continuous cardiac monitoring while undergoing diagnostic investigation. Laboratory exams revealed an absence of metabolic, endocrine, inflammatory, or myocardial injury changes (Table 1). Serology for chronic infections, as well as non-reagent exams, was conducted.

**Figure 1:** – 12-lead electrocardiogram showing complete atrioventricular block (AVB) with junctional escape rhythm and a heart rate of 30 bpm.

Source: Drafted by the author.

**Table 1:** – Laboratory exams and reference values. Source: Drafted by the author

Exams	Results	Reference Values
Hemoglobin	13.0 g/dL	13.5-17.5 g/dL
Leucocytes	10190 /mm^3^	3500-10500 /mm^3^
Eosinophils	41 /mm^3^	50-500 /mm^3^
Platelets	269000 /mm^3^	140000-440000 /mm^3^
Magnesium	2.04 mg/dL	1.6-2.6 mg/dL
Potassium	4.4 mg/dL	3.4-5.4 mg/dL
Sodium	143 mg/dL	135-147 mg/dL
Ionic Calcium	4.67 mg/dL	4.48-5.2 mg/dL
Ultrasensitive TSH	1.68 uUI/mL	0.27-4.20 uUI/mL
Ultrasensitive PCR	0.51 mg/dL	<1.0 mg/dL
Glucose	102 mg/dL	70-100 mg/dL
Arterial pH	7.44	7.35-7.45
Arterial HCO3	21 mmol/L	21-28 mmol/L
Arterial pCO2	32 mmHg	35-45 mmHg
Lactate	12.7 mg/dL	4.5-19.8 mg/dL
Creatinine	0.99 mg/dL	0.7-1.2 mg/dL
Urea	39 mg/dL	16-50 mg/dL
Ultrasensitive Tropinin T	9.77 pg/mL	< 14 pg/mL
Anti-HCV	NR*	NR*
Anti-HIV	NR*	NR*
HbsAg	NR*	NR*
Chagas	NR*	NR*
VDRL	NR*	NR*
FAN	NR*	NR*
Sars-CoV-2 antigen	NR*	NR*
Hydatidosis antibody (IgM/IgG)	NR*	NR*

* NR: non-reagent.

Once the potentially reversible causes had been discarded, we chose to perform a transthoracic echocardiogram (TTE), which showed an anechoic, cyst-like structure in the inner portion of the right atrium, attached to the interatrial septum and adjacent to the tricuspid septal leaflet, without causing diastolic eversion or obstruction in the right ventricle inflow tract. After the peripheral injection of a stirred saline solution, a more precise design of the structure was observed, without the filling of this structure or the passage of stirred solution among the chambers (Figure 2).

**Figure 2:** – Echocardiogram showing, in a 4-chamber apical window (A) and subcostal (B), anechoic, cyst-like structure in the inner portion of the right atrium, attached to the interatrial septum and adjacent to the tricuspid septal leaflet (red arrows). After peripheral injection of the stirred saline solution, a more precise design of the lesion was observed, with no filling of this by the solution (C and D). LA: left atrium; RA: right atrium; LV: left ventricle;RV: right ventricle.

Source: Drafted by the author.

Diagnostic hypotheses included the hydatid cyst or blood cyst. In a complementary manner, due to the unavailability of the magnetic resonance (MR), we chose to perform a computed tomography (CT) of the chest and abdomen to better characterize the lesion and its content. This action revealed a hypodense image of the regular and smooth contours, measuring 2.5 x 2.2 cm, near the basal region of the interventricular septum and the interatrial septum, in the right atrial cavity. The density was compatible with the liquid content, which was most likely not related to the blood, even after the injection of contrast. The CT of the abdomen identified no cysts in other organs (Figure 3).

**Figure 3:** – Computed chest angiotomography in an axial cut, showing the hypodense image of regular and smooth contours, measuring 2.5 x 2.2 cm, with liquid content, located near the interatrial region and basal portion of the interventricular septum.

Source: Drafted by the author.

Despite the negative serology by indirect immunofluorescence, the environmental exposure and the imaging exams reinforce the hypothesis of the hydatid cyst, as well as the fact that this is the cause of the conduction disorder secondary to the extrinsic compression of the AV node. Due to the patient’s refusal to undergo surgery during hospitalization, even though he was aware of the risks, we chose, together with the family, to perform a definitive pacemaker implant, with subsequent hospital release with antiparasite treatment. After three months of outpatient follow-up, the TTE was repeated, which revealed a discrete reduction in the size of the cyst when compared to the pre-treatment medication image (Figure 4), with no signs of complication; the patient remained asymptomatic.

**Figure 4:** – Transthoracic echcardiogram showing the cystic image with a discrete reduction in the dimensions when compared to the previous exam, measuring approximately 2.1 x 2.0 cm.

Source: Drafted by the author.

## Discussion

Hydatidosis, or echinococcosis, is a parasite infection generally caused by *Echinococcus granulosus*, which occurs in an endemic manner in some regions in the world. When in a larval stage, the parasite can infect humans in an accidental manner through the intake of contaminated food, in which dogs and other carnivores are definitive hosts, and omnivores and herbivores are intermediate hosts.^1^

The infection commonly results in the formation of hepatic and pulmonary cysts, since the hematological dissemination occurs by means of absorption through the gastrointestinal tract and consequent invasion of the portal circulation and inferior vena cava.^3^ The involvement of other organs is less common, with cardiac involvement being extremely uncommon (0.5-2% of the cases). Left ventricular involvement is more commonly observed (60%), followed by right ventricular (10%), pericaridial (7%), left atrium (6-8%), and interventricular septum (4%).^4^ The right atrium, as described in the present case report, is only involved in 3-4% of the cases.^3^

Clinical manifestations depend on the location, growth, and number of cysts. They can appear with unspecific symptoms (cough, fever, weight loss, fatigue, chest pain) or, in most cases, in an asymptomatic form.^5^ Hydatidosis is associated with complications, such as arrythmias, pulmonary or systemic embolic events, and anaphylactic shock secondary to rupture.^3^ The growth of the cyst can cause complications due to extrinsic compression, such as myocardial ischemia, conduction disorders, valve dysfunction, or obstruction of the transvalvular outflow.^1, 6^

The diagnosis is performed through the combination of clinical findings, serological tests, and imaging exams.^3^ The ELISA test is one of the most sensitive in detecting antibodies against *Echinococcus granulosus*. However, one can also use indirect immunofluorescent tests, complement fixation through enzymatic immunoassay (Weinberg), and the latex agglutination test.^3,6^ Negative tests do not exclude the diagnosis, since the detection would be greater in cases of hematogenic dissemination secondary to the release of the cyst’s content. With this, imaging methods become essential tools for their definition.^6^

The TTE is a more effective method in the diagnosis due to its easy access and low cost. The cyst presents a hypoechogenic component and a regular contour, and enables a detailed characterization regarding the size, quantity, location, and identification of complications.^1^ MR shows greater precision when evaluating the cyst’s content and its relation with adjacent structures.^3^ The aspect is of an oval, hypointense lesion in the weighted images in T1 and hyperintense in T2. The typical finding is a hypointense peripheral ring, representing a pericyst (dense fibrous capsule of the reactive host tissue).^4^ By contrast, the CT exam revealed a hypodense structure with the accumulation of contrast in the walls.^6^ An uncomplicated, hydatid cyst, as in the present case report, shows a well-defined homogenous lesion, with low density and smooth walls, with variable thickness.^7^ More specific imaging findings include calcification of the cyst wall, new cysts, and detachment of the membrane.^3,4,7^

As regards the differential diagnosis, one can consider cardiac tumors as myxomas, blood cysts, and congenital pericardial cysts.^4,7^ In the present case report, the cystic lesion was identified by the TTE and itsa analysis was complemented by CT exam, with hydatidosis being the main hypothesis.

Surgical removal is the treatment of choice, even in asymptomatic patients due to the high risk of rupture, requiring a cardiopulmonary bypass to avoid dissemination.^6,8^ After removal, the treatment of choice is albendazole (10-15 mg/kg/day) for 3 to 6 months.^6,9^ Patients who are not candidates for invasive treatment should use anthelmintic drugs to reduce the growth of the cyst.^6^ The pre-operatory treatment tends not to be recommended due to the increase in the friability of the membrane, thereby augmenting the risk of rupture during surgery.^8^

The postoperative prognosis is good, in which worse complications are rare.^6,10^ Data is lacking regarding cases not submitted to surgical procedures. Although the procedure is considered to be curative in an isolated cardiac form, it does present a risk of recurrence, requiring long-term follow-up.^6^

## Conclusion

Hydatidosis is an endemic zoonosis with a rare cardiac involvement. The present study reports on a case of hydatic cyst, with an uncommon location and atypical clinical presentation, leading to a complete AVB in a young adult patient. The etiological diagnosis in this scenario should undergo thorough investigation, with the imaging exams being essential to determine the appropriate diagnostic handling and therapeutic definition.
